# Interchangeability of Two 500 Mg Amoxicillin Capsules with One 1000 Mg Amoxicillin Tablet After a Single Oral Administration

**DOI:** 10.4103/0250-474X.73904

**Published:** 2010

**Authors:** A. N. Zaid, R. Cortesi, J. Kort, W. Sweileh

**Affiliations:** College of Pharmacy, An-Najah National University, Nablus, Palestine; 1 Department of Pharmaceutical Sciences, Ferrara University, Ferrara; 2 Pharmacare Chemical and Cosmetics, Ramallah, Palestine

**Keywords:** Amoxicillin, bioequivalence, capsules, interchangeability, pharmaceutical formulation, tablets

## Abstract

The aim of the study was to evaluate if two capsules (Amoxil^®^ capsules, 500 mg/capsule) and one tablet (Amoxicare^®^ tablets, 1000 mg/tablet) of amoxicillin have similar bioequivalence parameters. For this purpose a randomized, two-way, crossover, bioequivalence study was performed in 24 healthy, male volunteers, divided into two groups of 12 subjects each. One group was treated with the reference standard (Amoxil^®^) and the other one with the generic tablet Amoxicare^®^, with a crossover after a wash-out period of 7 days. Blood samples were collected at fixed time intervals and amoxicillin was determined by a validated HPLC method. The pharmacokinetic parameters AUC_0-8_, AUC_0-∞_, C_max_, T_max_, K_e_ and T_1/2_ were determined for both formulations and statistically compared to evaluate the bioequivalence between the two brands of amoxicillin, using the statistical model recommended by the FDA. C_max_ and AUC_0-∞_ were statistically analyzed using analysis of variance (ANOVA); no statistically significant difference was observed between the two formulations. The 90% confidence intervals between the mean values of C_max_ and AUC_0-∞_ fall within the FDA specified bioequivalent limits (80-125%) suggesting that the two products are bioequivalent and the two formulations are interchangeable. Based on these findings it was concluded that the practice of interchangeability between the above formulations to achieve better patient compliance could be followed without compromising the extent of amoxicillin absorption.

Amoxicillin (C_16_H_19_N_3_O_5_S; Mol. Wt: 365.41; λ_max_: 230-273.8 nm, ethanol; LogP: 0.91; fig. 1) is a commonly prescribed orally available β-lactam antibiotic with moderate antibacterial spectrum that is available on the market as different dosage forms and in strengths. For instance, it is available as 1000 mg tablet and as 500 mg capsules. The question of whether two 500 mg capsule can be substituted with one 1000 mg tablet is not uncommon and pharmacists face such problem frequently with many other prescribed medications. Possible reasons for substitution include the following; (i) the prescribed original brand or the dosage is not available in pharmacy as the case in many developing countries, (ii) difference in the price of the prescribed dosage form and (iii) better swallowing characteristics especially for elderly patients. The later reason is of particular importance as swallowing problems can cause poor patient compliance. In fact, some studies have indicated that some dosage forms are preferred over others by elderly patients. For example, capsules are preferred over tablets when large amount of active ingredient is to be swallowed[1]. In fact, the rugged surface of some tablets may scratch the esophagus during intake[2]. Indeed, damage of esophagus by retention of tablet has been reported[3]. Furthermore, some tablets have strong smell or taste. All these factors may make the administration of tablets an uncomfortable experience leading to poor patient compliance. In such cases, substitution of large volume tablet with two capsules containing the same drug strength can solve problems associated with swallow ability. However, bioequivalence problems may arise especially when the substitution was carried out between liquid and solid oral dosage forms, or between soft elastic gelatin capsules and tablets[4]. On the other hand, bioequivalence cannot be compromised when two solid dosage forms such as hard gelatin capsules and tablets where interchanged. Few publications are found in literature demonstrating potential interchangeability of two different solid dosage forms based on pharmacokinetic data[5,6]. The aim of the present study was to determine if the practice of interchangeability between two amoxicillin capsules (Amoxil^®^, 500 mg/capsule) and one amoxicillin tablet (Amoxicare^®^, 1000 mg/tablet) in order to achieve better patient compliance can be conducted without compromising their extent of absorption

**Fig. 1:**
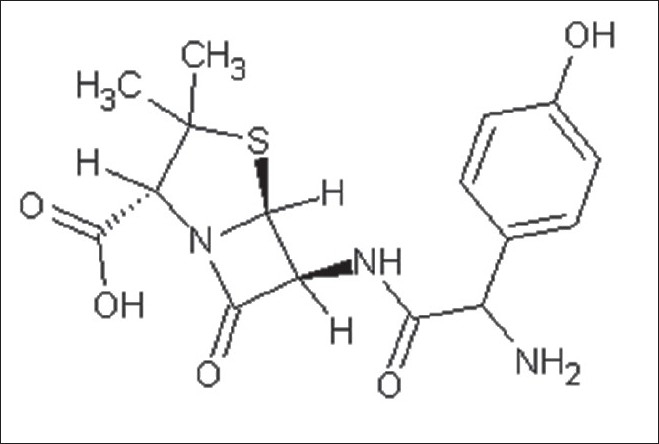
Chemical structure of amoxicillin

## MATERIALS AND METHODS

Acetonitrile HPLC grade, methanol HPLC grade and tetra-n-butyl ammonium hydroxide were from Merck, (Darmstadt, Germany); chloroform HPLC grade was from Fisons Scientific Equipments, UK; sodium dihydrogen phosphate dihydrate (NaH_2_PO_4_·2H_2_O) was from Riedel de Haën, (Seelze, Germany); double distilled high purity water was used. The HPLC grade solvents acetonitrile and methanol were used as received. All other reagents were of analytical grade. Amoxicillin and Paracetamol (internal standard) were supplied by Pharmacare Chemical and Cosmetics (Ramallah, Palestine). Amoxicare^®^ tablet (1000 mg amoxicillin/tablet, batch number RD-23E03) was from Pharmacare Chemical and Cosmetics. Amoxil^®^ capsules (500 mg/capsule, batch number 040605) were manufactured by Medical Union Pharmaceuticals Co, Abou Sultan Egypt, under license of Beecham Pharmaceuticals Brantford England.

The protocol of the study was approved by the Ethical Committee of Tanta University Hospital (Tanta, Egypt). The whole study followed the requirements of the Declarations of Helsinki and was conducted in accordance with the current Good Clinical Practice (GCP), International Conference Harmonization (ICH) as well as Good Laboratory Practice (GLP) Guidelines. Each volunteer had signed the informed consent form after ample information and consideration time has been provided.

Twenty-four adult male volunteers, non-smokers, aged between 17-30 y, weighing between 59 and 85 kg, were chosen to participate in the present study. The volunteers were not on concomitant medications and they were free from significant cardiac, hepatic, renal, pulmonary, gastrointestinal, neurological or hematological disease as determined within four weeks prior to the beginning of the study by way of medical histories and physical examinations. Subject’s health status was determined following a physical examination, laboratory tests and medical history by a qualified registered MD physician. The physician reviewed all preclinical laboratory tests for each subject. Exclusion criteria included extreme weight ranges (overweight or underweight), anemia, liver or renal dysfunction, parasitic and other diseases or conditions that was judged to affect the absorption, distribution and/or elimination of amoxicillin. The subjects were asked to abstain from taking drugs and alcohol for at least 3 days prior to the test and throughout the entire study period. However, it has to be underlined that the drugs eventually used by the involved volunteers are characterized by short half life values and thus do not overlap with amoxicillin during the first day of study. On the night before starting the study, the volunteers were instructed to fast for at least 10 h before drug administration. The study had an open randomized two-period crossover design with a 7-day washout period between doses. The volunteers were randomly divided into two equal groups each of 12 subjects. The first group was given the reference formulation and the second group received the test formulation with a crossover after a washout period of one week. On the morning of the experiment, 10 ml of blood sample was withdrawn from each volunteer to serve as a blank for the drug assay. Each of the 24 volunteers then took one Amoxicare^®^ tablet (1000 mg amoxicillin) as the test drug or two Amoxil^®^ capsules (500 mg amoxicillin each) as the reference drug followed by 200 ml of water. Five milliliters blood samples for plasma drug assay were collected from an indwelling catheter inserted in the antecubital vein of one of the arms. Samples were obtained at 0.0 (blank sample), 0.25, 0.5, 0.75, 1, 1.5, 2, 2.5, 3, 4, 6, 8 h after drug administration, in heparinized tubes. Plasma was directly separated by centrifugation at 3000 rpm for 10 min and stored at -20° until analyzed. Four hours after drug administration, the subjects were allowed to eat a standard breakfast of bread, jam, low-fat white cheese, water (150 ml). They were then allowed controlled access to fruit juice and other non-alcoholic beverages. The volunteers had their second meal (standard lunch containing grilled chicken, rice and vegetables) 4 h later.

Stock solution of amoxicillin (500 µg/ml) was prepared by dissolving 50 mg of drug in methanol. Working standard solutions were prepared from the stock solution by serial dilution with methanol to prepare seven working solutions of amoxicillin with concentrations of 5, 10, 20, 50, 100, 200 and 500 µg/ml. Stock solution of paracetamol (internal standard) was prepared by dissolving the drug powder in methanol to give 50 µg/ml. Stock and working standard solutions were protected from light and stored at -20° until use. The calibration standards were prepared by adding known amounts (50 µl) of amoxicillin to each working solution, and 50 µl of the internal standard solution to a set of clean test tubes. After evaporation of the methanol solution 0.5 ml of blank plasma was added to each tube to form a set of calibration standards with concentrations of 0.5, 1.0, 2.0, 5.0, 10, 20 and 50 µg/ml.

The calibration standards were treated with 1 ml of acetonitrile and shaken for 1 min, then centrifuged at 3000 rpm for 7 min. The supernatant was transfered to clean test tube and 5 ml of chloroform was added, shacked for one minute, and then centrifuged at 3000 rpm for 7 min. The upper layer was separated and 50 µl was injected into the HPLC column. For the analysis of the samples, 0.5 ml of each study sample was transferred to a clean test tube spiked with the internal standard. The study samples were treated as the calibration standards after addition of the internal standard. Under the chromatographic conditions described above, the retention time of amoxicillin and the internal standard (paracetamol) were 4.8±0.5 and 6.0±0.5 min, respectively. Analytical validation report for HPLC method used for the quantification of amoxicillin in human plasma is described below.

The analyses were performed using an HPLC system equipped with a variable wavelength UV detector and an automatic injector was used in this study Waters 2690 Separation Modules, Waters Corporation, Milford Massachusetts, USA. Separation was accomplished with a 250×4.6 mm 5 µm Hypersil^®^ BDS C_18_ column, Thermo Fisher Scientific Inc, Waltham, United States. The mobile phase used consists of 94% buffer and 6% acetonitrile, where buffer is a mixture of tetra-n-butyl ammonium hydroxide (0.15 g/l) in sodium dihydrogen phosphate dehydrate (20 mM). The flow rate was 1.2 ml/min, the UV detector was set at 225 nm and the peak areas were calculated using the Millennium data analysis program (Millennium Consultants Inc, NJ, USA). Quantification of amoxicillin was obtained by plotting amoxicillin to internal standard peak area ratios as a function of concentration. For statistical analysis a Minitab statistical Package version 13 on IBM PC was used.

The validation of this chromatographic analytical method was performed in order to evaluate its linearity, selectivity, stability, precision and accuracy. The obtained data were summarized in Table 1. Calibration curves were constructed from the peak area ratio (drug/ internal standard) and the corresponding amoxicillin concentration in each calibration standard. The linearity study was carried out in the range of concentrations between 0.5 to 50 µg/ml. To assess linearity, drug free plasma was spiked with known amounts of the drug to achieve the concentration of 0.5, 1.0, 2.0, 5.0, 10, 20 and 50 µg/ml. The correlation coefficient was always greater than 0.99 during the course of the validation.

**TABLE 1:** METHOD VALIDATION PARAMETERS OF AMOXICILLIN ANALYSIS

Parameters	Results
Linearity range	0.5-50 mg/ml
Correlation coefficient	0.9978
Regression equation (y=mx+q)	
m	0.208
c	0.056
Limit of quantitation (LOQ)	0.5 µg/ml
Coefficient of variation intraday (%CVintra) (n=3)	from 0.735 to 13.38%
Coefficient of variation inter-day (% CVinter) (n=3)	from 0.783 to 13.13%
Accuracy intra-day (%)	from 95 to 109%
Accuracy inter-day (%)	from 93 to 112%

Precision was determined as the coefficient of variation (CV), and the accuracy as the percentage relative error (RE) of a series of measurements. Precision and accuracy data were obtained by analyzing aliquots of three-spiked plasma at low middle and high concentration levels of amoxicillin. Intraday reproducibility was determined by analyzing three replicates of calibration curves on the same day, and inter-day reproducibility was evaluated by the analysis of six different calibration curves on six different days during the study period. The intraday coefficient of variation and the inter-day coefficient of variation were within 10% indicating that the method is precise. The accuracy of the method was validated as the intra-day accuracy was in the range from 95 to 109% and the inter-day accuracy was in the range of 93 to 109% during the entire period in which calibration curves were generated.

The drug stability at room temperature was determined preparing three different plasma samples of each drug concentration and then injecting the samples immediately into the HPLC system. The samples were kept at room temperature and were injected again after 8 h. The concentrations measured at time zero and after 8 h were compared to determine whether there were changes in the concentrations with time. The difference in the drug concentrations in the two analyses was always less than 10%. When the samples were kept at room temperature for 8 h, no changes in the drug concentration was evident thus indicating the stability of amoxicillin in the plasma samples at room temperature.

The stability of the drug in frozen plasma was investigated by the analysis of samples obtained from three volunteers. The first analysis was performed at the beginning of the study and the second analysis was performed at the end of the study. The samples were stored at -20° C between the analyses. The difference in the drug concentration in all samples in the two analyses was always less than 10% in each sample. There was no change in the drug concentration in frozen plasma during storage at -20° during the study period, indicating stability of amoxicillin in frozen plasma.

The lower limit of quantification (LLOQ) was estimated by analyzing samples with known amounts of amoxicillin, at progressively lower concentrations, starting at the lower end of the calibration curves. The limit of quantification of amoxicillin in this assay was 0.5 µg/ml. The retention time of the drug in the standard and the study samples were identical. There were no peaks for endogenous compounds that appeared at the same retention time for amoxicillin in the chromatograms for six different blank plasma samples.

The concentration of amoxicillin in the plasma (µg/ml) at different time points following administration of either one Amoxicare^®^ tablet (1000 mg amoxicillin) or two Amoxil^®^ (500 mg amoxicillin each) were plotted for each volunteer. Parameters such as C_max_ and t_max_ were recorded. The area under the curve of plasma drug concentration (AUC) versus time (t), from zero to t (AUC_0→t_), was calculated from the drug concentration by the linear trapezoidal rule, using the relationship;

1 $${\mathrm{AUC}}_{0\to t}\;=\;\Sigma \frac{\left(C_{n}\;+\;C_{n+1}\right)}{2}x\left(t_{n+1}\;–\;t_{n}\right)$$

where, C_n_ is the drug concentration at any time (t_n_). The area under the curve from t h to infinity was obtained from the relationship:

2 $${\mathrm{AUC}}_{t\to \infty }\;=\;C_{t}\;/\;K_{e}$$

where, C_t_ is the concentration of amoxicillin at the t-hour time point (last determined concentration), K_e_ is the elimination rate constant in a particular individual. The elimination rate constant (K_e_) was calculated from the slope of the straight part of log C versus t plot, where the slope of the straight line equals (-k/2.303). To obtain this value, the data were plotted as log plasma concentration versus time (to ensure linearity of the terminal phase of the profile) and the best fitting line was determined by the method of least square. The total area under the curve from zero to infinity (AUC_0→∞_) was calculated as the sum of the areas obtained

3 $${\mathrm{AUC}}_{0\to \infty }\;=\;{\mathrm{AUC}}_{0\to t}\;+\;{\mathrm{AUC}}_{t\to \infty }$$.

Following the oral administration of drugs, the plasma concentration generally reaches a single, well-defined peak (C_max_) at the time of T_max_. C_max_ is an important kinetic index for drug safety and can be viewed as a measure of maximal exposure. Particularly, C_max_ has important roles in clinical pharmacological investigations, including bioequivalence, T_max_ has been considered as a simple measure for comparative absorption and disposition rate constants. C_max_ and T_max_ can be estimated either directly or indirectly. In the first case, one can record the maximal observed concentration and the corresponding time. In the second case, a pharmacokinetic model can be fitted to the measurements and the predicted maximum evaluated[7]. The maximum amoxicillin plasma concentration (C_max_) and the corresponding time of peak plasma concentration (T_max_) were taken directly from the slope of the semi-logarithmic plot of the terminal phase of the plasma concentration-time curve calculated by linear regression. The elimination half–life (T_1/2_) was derived by dividing ln2 by the elimination rate constant K_e_. The areas under the amoxicillin plasma concentrations time curves from (AUC_0-8_) and the area to the infinity (AUC_0-∞_) were calculated by using the linear trapezoidal method. Extrapolation to infinity was obtained by adding the value C_t_/K_e_ to the calculated AUC_0-8_ (where C_t_ is the last detectable concentration of amoxicillin). For the purpose of bioequivalence analysis, one-way analysis of variance (ANOVA procedure) was used to assess the effect of formulations, periods, sequences and subjects on AUC_0-8_, AUC_0-∞_, and C_max_. The statistical analysis was performed using Minitab Statistical package version 13 IBM PC (Minitab Inc. Quality Plaza USA).

## RESULTS AND DISCUSSION

**Fig. 2:**
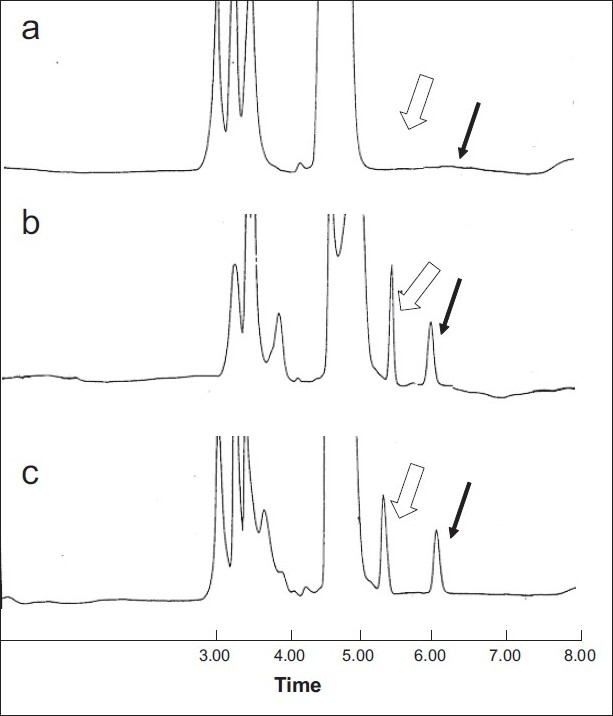
Representative chromatograms of amoxicillin. Panel (a) Blank plasma sample. Panel (b) Blank plasma spiked with amoxicillin (20 µg/ml) and internal standard (I.S.). Panel (c) Plasma sample of volunteer #14R obtained after 1.5 hr from administration. Internal Standard: closed arrow. Amoxicillin: empty arrow.

**Fig. 3:**
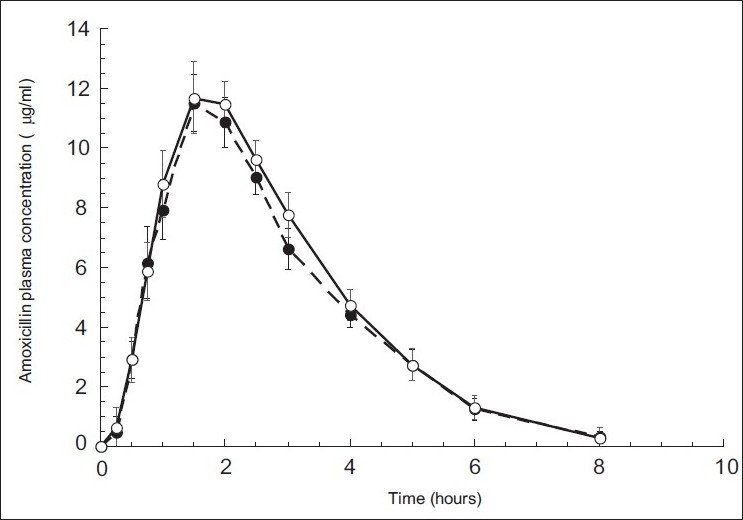
Mean plasma concentration versus time plots of amoxicillin after oral administration. Oral administration was performed on 24 volunteers using Amoxil® (closed circles) as test formulation or Amoxicare® (open circles) as reference.

The alternate HPLC-UV method described and used here for amoxicillin quantification provided the required sensitivity, specificity and high sample throughput required for pharmacokinetic studies. In fig. 2 are shown three representative chromatograms of amoxicillin. Particularly, blank plasma sample chromatogram, blank plasma spiked with amoxicillin (20 µg/ml) chromatogram and plasma sample of volunteer #14R obtained after 1.5 h from administration, are reported. Both amoxicillin formulations (one Amoxicare^®^ 1000 mg/tablet, and two Amoxil^®^ 500 mg/capsule) were well tolerated at the administered dose by all the subjects; unexpected incidents possibly influencing the outcome of the study did not occur. All volunteers were discharged from the hospital in good health conditions. Both medications were quantifiable at the first sampling time in all the volunteers. fig. 3 shows mean amoxicillin plasma concentrations as a function of time after the oral administration of 1000 mg of amoxicillin of both brands over the 8 h truncated sampling period. Fig. 3 shows that both formulations were matching in terms of plasma drug concentration - time curves. Detailed descriptive statistics of the major mean pharmacokinetic parameters including AUC0_-8_, AUC_0-∞_, C_max_, T_max_, K_e_ and T_1/2_ for the test and reference formulations are summarized in Table 2

**TABLE 2:** PHARMACOKINETIC PARAMETERS CALCULATED FOR AMOXICILLIN AFTER A SINGLE ORAL DOSE ADMINISTRATION*

	C_max_ (µg/ml)	T_max_ (h)	K_e_ (h-1)	T _½_ (h)	AUC_0-8_ (µg×h/ml)	AUC_t-∞_ (µg×h/ml)	Total AUC_0-.∞_(µg×h/ml)
Mean (Amoxicare^®^)	14.27	1.677	0.616	1.269	36.83	2.189	39.02
SD (Amoxicare^®^)	5.693	0.502	0.226	0.436	11.39	1.582	11.73
CV% (Amoxicare^®^)	0.399	0.299	0.366	0.343	0.309	0.722	0.307
MIN (Amoxicare^®^)	4.039	0.750	0.340	0.612	11.85	0.556	15.88
MAX (Amoxicare^®^)	33.51	3.000	1.131	2.035	58.76	6.594	60.09
Mean (Amoxil^®^)	13.24	1.750	0.603	1.245	34.75	1.967	36.72
SD (Amoxil^®^)	4.319	0.436	0.164	0.392	11.29	1.289	11.74
CV% (Amoxil^®^)	0.326	0.249	0.272	0.315	0.325	0.655	0.319
MIN (Amoxil^®^)	5.359	0.750	0.306	0.688	12.690	0.551	14.71
MAX (Amoxil^®^)	20.51	2.500	1.006	2.260	53.750	5.312	55.201

* Oral administration was performed on 24 healthy male volunteers using one 1000 mg tablet of Amoxicare^®^ (test formulation) or two 500 mg capsules of Amoxil^®^ (reference formulation).

The relative bioavailability of amoxicillin from test formulation (one Amoxicare^®^ tablet containing 1000 mg amoxicillin) compared to the reference formulation (two Amoxil^®^ capsules containing 500 mg amoxicillin/capsule) was found to be 112.28% of AUC_0-8_, 106.26% of AUC_0-∞_ and 107.78% of C_max_. Moreover, the parametric 90% confidence intervals for those pharmacokinetic parameters values were entirely within the FDA specified bioequivalent limit (80-125%). Exactly, the 90% confidence limit of the mean values of C_max_ and AUC_0-∞_ for Amoxicare^®^ tablets and Amoxil^®^ capsules fall between (99.18-107.2%) and (99.58-107.6%) of the reference mean, for C_max_ and AUC_0-∞_, respectively. These statistical results of ANOVA parameters indicate that the two products are bioequivalent and so interchangeable. Based on the results mentioned above, interchangeability between Amoxicare^®^ 1000 mg tablet and two Amoxil^®^ 500 mg capsules can be performed.

Two formulations of the same drug are considered to be bioequivalent and *per se* therapeutically equivalent if they exhibit a comparable extent and rate of absorption, when they are administered in the same molar dose and under similar experimental conditions[7–9]. AUC_0-∞_, C_max_ and T_max_ values were statistically analyzed for determination of bioequivalence. Other pharmacokinetic parameters (i.e., T_1/2_ and K_e_) results were used as supporting results in this pharmacokinetic design. Statistical comparison of the main pharmacokinetic parameters, AUC_0-8_, AUC_0-∞_, C_max_ and T_max_ clearly indicated no significant difference between test and reference in any of the calculated pharmacokinetic parameters. The obtained values were in good agreement with the FDA requirements for bioequivalence of generic drugs[10]. Since the AUC_0-∞_ and Cmax mean ratios are within the 80–125% interval, it was concluded that the tested formulation Amoxicare^®^ elaborated by Pharmacare, Palestine, is bioequivalent for both extent and rate of absorption to two commercial Amoxil^®^ capsule manufactured by Medical Union Pharmaceuticals Co, Abou Sultan, Egypt, under license of Beecham Pharmaceuticals, Brantford, England. These findings may be considered as further useful demonstration regarding the safety and efficacy of interchanging of one tablet containing 1000 mg with two capsules containing 500 mg each one. Furthermore, this study should help health practitioners in convincing patients toward interchanging large tablets with two capsules of smaller size to improve patient compliance when swallowing problems obstacles the regular and appropriate drug administration. Indeed, if a patient has once experienced a tablet sticking on esophagus, the patient may get unpleasant association by swallowing tablets thereafter. Thus taking two capsules containing the same strength of pharmaceutical active ingredient present in one tablet should be convenient and easy task. In fact, a recent study has shown that 100 mg and 400 mg imatinib tablets were comparable, in terms of bioequivalence, safety, and tolerability to the commercial 100 and 400 mg imatinib hard gelatin capsules[5]. In another similar study two saquinavir 500 mg film coated tablets were bioequivalent to five saquinavir 200 mg hard gelatin capsules[6]. Since the substitution of one amoxicillin 1000 mg tablet with two 500 mg amoxicillin capsules or vice versa is a common practice, the objective of this study was to demonstrate that this substitution does not compromise the bioequivalence parameters.

The validated HPLC method employed here proved to be simple, fast, reliable and selective enough to be used in clinical pharmacokinetic studies of amoxicillin in humans. The results showed that both test formulation (Amoxicare^®^, 1000 mg amoxicillin/tablet) and reference formulation (Amoxil^®^, 500 mg amoxicillin/capsule) were bioequivalent, since both deliver equivalent amounts of amoxicillin to the systemic circulation at equivalent rates. This suggests that interchangeability between these two oral solid dosage forms can be performed provided that the administered amount of active ingredient in the test and reference formulation is equal. Finally, further studies are required in order to extrapolate and expand the results of these studies to other medications and dosage forms.
